# Applying the RE-AIM implementation framework to evaluate diabetes health coaching in individuals with type 2 diabetes: A systematic review and secondary analysis

**DOI:** 10.3389/fendo.2022.1069436

**Published:** 2022-12-13

**Authors:** Megan Racey, Milos Jovkovic, Paige Alliston, Diana Sherifali

**Affiliations:** ^1^ McMaster Evidence Review and Synthesis Team, McMaster University, Hamilton, ON, Canada; ^2^ School of Nursing, Faculty of Health Sciences, McMaster University, Hamilton, ON, Canada

**Keywords:** health coaching, systematic review, RE-AIM (reach, effectiveness, adoption, implementation and maintenance), type 2 diabetes

## Abstract

**Background:**

Diabetes health coaching continues to emerge as an effective intervention to support diabetes self-management. While previous systematic reviews have focused on the effectiveness of diabetes health coaching programs in adults with type 2 diabetes (T2DM), limited literature is available on its implementation. This review examines what aspects of diabetes health coaching interventions for adults living with type 2 diabetes have been reported using the Reach, Effectiveness, Adoption, Implementation, and Maintenance (RE-AIM) framework to optimize implementation.

**Methods:**

We examined the included studies from our recently completed systematic review, which searched 6 databases for randomized controlled trials (RCTs) of health coaching interventions delivered by a health professional for adults with T2DM. Reviewers screened citations and extracted data for study characteristics and the 5 dimensions (62 criteria) of the RE-AIM framework.

**Results:**

9 diabetes health coaching RCTs were included in this review. 12 criteria were reported by all the included studies and 21 criteria were not reported by any of the studies. The included studies all reported on more than 20 RE-AIM criteria, ranging from 21 to 27. While Reach was the best reported construct by the included studies, followed by Effectiveness and Implementation, the criteria within the Adoption and Maintenance constructs were rarely mentioned by these studies. In general, there was also wide variation in how each of the criteria were reported on by study authors

**Conclusions:**

Due to the paucity of reporting of the RE-AIM components for diabetes health coaching, limited implementation and clinical practice implications can be drawn. The lack of detail regarding implementation approaches to diabetes health coaching greatly limits the interpretation and comparisons across studies to best inform the application of this intervention to support diabetes self-management.

**Systematic review registration:**

PROSPERO identifier, CRD42022347478

## 1 Introduction

Individuals living with type 2 diabetes (T2DM) are responsible for the majority of their self-management, spending only very limited time with their healthcare providers while the remaining time spent on self-management is completed by the individual outside of the healthcare setting. However, one’s ability to self-manage chronic illnesses is dependent on several factors, including sociodemographic variables (e.g. income, culture, literacy, environment), behavioural considerations (e.g. eating and activity habits), and comorbidities (1). Despite the availability of diabetes education programs, engagement with such programs has been challenged by a) limited availability, offerings, and duration of education, support, and specialized programming and b) minimal individualized or tailored education and support (2–5).

Diabetes health coaching is increasingly viewed as an effective strategy to support self-management. According to Wolever et al., health coaching may be described as: a) patient centred; b) includes patient determined goals; c) incorporates self-discovery and active learning processes; d) encourages accountability for behavioural goals; e) provides some education alongside coaching; f) a health professional who is trained in behaviour change, communication, and motivational interviewing skills (6). Health coaching may also be timely and relevant health related education, behaviour change promotion, and psychosocial support to enhance the well-being of individuals and facilitate the achievement of their health-related goals (7, 8). More recently, health coaching models have been proposed to help describe and define these interventions (9). This model is comprised of four components: (i) personal case management and monitoring, emphasizing process of care issues and system navigation related to diabetes; (ii) diabetes self-management education and support, highlighting the need for knowledge, skill acquisition, and problem solving related to day-today management; (iii) behaviour modification, goal setting and reinforcement, using motivational interviewing and theories to facilitate goal setting, attainment, and behaviour change; and (iv) general psychosocial support, leveraging active listening and empathy to provide support. Any of these components may be involved in health coaching programs.

Several reviews show a consistent statistically significant reduction in glycated hemoglobin (A1C) of approximately 0.24% to 0.66% with exposure to a diabetes health coach (10–12). But despite the rapid interest in this diabetes health coaching, the description of the role of coaches and how these interventions are implemented and evaluated remains limited. Moreover, the implementation (e.g. training, delivery) and the short and long term evaluation measures related to diabetes health coaching has not been fully described and reported in the literature (9). A previous review conducted in 2015 found that although eight trials reported effectiveness on glycemic control, details of the implementation and evaluation of diabetes health coaching were limited and mainly pertained to the specific training requirements of health care professionals (11).

Regardless of the availability of many implementation theories, checklists, and strategies, to date, no implementation frameworks have been applied to the diabetes health coaching literature, with only scant discussions related to implementation in the literature. The Reach, Effectiveness, Adoption, Implementation, and Maintenance (RE-AIM) framework was created to improve the transparency in reporting of the essential components of an intervention, with the goal of ultimately supporting the adoption and implementation of evidence-based interventions (13). Although the RE-AIM framework is generally used as a planning tool for scaling up and sustaining the spread of interventions, it has not been applied to the diabetes health coaching literature to date.

Therefore, the application of the RE-AIM framework to diabetes health coaching intervention components will further elucidate the critical aspects of the intervention to ensure the adoption, scaling, and maintenance of an intervention that is effective in supporting diabetes self-management support. Leveraging the findings of a recently completed systematic review and meta-analysis on the effectiveness of diabetes health coaching trials by Racey et al., the goal of this systematic review is to examine the application and reporting of the RE-AIM components in the included studies, which will inform the feasibility and scalability of future diabetes health coaching work.

## 2 Methods

This review is a secondary research question to a systematic review and meta-analysis (12). This paper examines the implementation components of health coaching interventions in adults with T2DM from the registered protocol (PROSPERO-CRD42022347478).

### 2.1 Search strategy

The search terms, databases, and strategy were developed in consultation with a research librarian at McMaster University and informed by a previous systematic review (11) (**Supplemental Material 1**). We searched MEDLINE, Embase/Emcare, Cumulative Index of Nursing and Allied Health Literature (CINAHL), PsycINFO, Cochrane Database of Systematic Reviews (CDSR) and Cochrane Central Register of Controlled Trials (CENTRAL) from inception to December 2021. We manually searched reference lists of relevant reviews and included studies for citations that were not captured in our search. Results from the search were deduplicated, and citations were uploaded to a secure internet-based platform for screening (DistillerSR, Evidence Partners Inc., Ottawa, Canada).

### 2.2 Study selection and eligibility

The eligibility criteria were established for the primary systematic review and have been previously explained (12). Briefly, studies had to be written in English, been published in a peer-reviewed journal, and meet the following criteria: 1) be a randomized controlled trial at the patient-level; 2) report data on adults ≥18 years of age with T2DM; 3) be a health coaching intervention (beyond one-dimensional education programs and including components as defined by Wolever et al. and Sherifali et al.) that was delivered, led, and/or implemented by a regulated healthcare professional, one who would routinely see patients with diabetes for care or management in a healthcare setting such as a clinician, nurse, or diabetes educator in primary care, community care, or hospital-based programs; and 4) include a control group which was defined as treatment as usual, standard care, or minimal contact that did not contain intervention components. Outcomes were not used for inclusion or exclusion of the studies. Studies were excluded if: 1) they reported data on participants younger than 18 years of age, who did not have type 2 diabetes, or who were pregnant; 2) health coaching was not the primary intervention; and 3) they were not randomized controlled trials, used a quasi-randomization methodology, including cluster randomization, or were pilot or feasibility trials.

### 2.3 Data extraction and quality assessment

A team of researchers conducted the screening and data extraction (MR, MJ, PA, DS). A minimum of two reviewers were required to independently and in duplicate screen titles and abstracts of all potentially eligible studies. Articles marked for inclusion by either team member went on to full-text screening which was completed independently and in duplicate by 2 team members and required consensus for inclusion or exclusion. We developed, piloted, and deployed standardized forms for data extraction. All relevant data was extracted using standardized forms. For each study, one team member extracted study characteristics and the 5 dimensions (62 criteria) of RE-AIM (13, 14) and a different team member verified the extraction. Studies were assessed for Risk of Bias in our complementary review (12). All conflicts for screening and data extraction were resolved by the lead researcher of this review (M.R.).

For the RE-AIM data extraction, reviewers used an adapted extraction tool designed specifically for conducting systematic reviews using RE-AIM (14). The tool outlined each RE-AIM criteria and their definitions for consistent extraction of each component. Reach was evaluated by 12 criteria including descriptions of the target population, inclusion, and exclusion criteria, who participated or was exposed to the intervention, participation rates, and characteristics of those who participated and those who did not. Effectiveness (or efficacy) was evaluated by 9 RE-AIM criteria including reporting of mediators and moderators, how data were treated, quality of life, unintended or negative consequences, and attrition. Adoption was assessed at both the setting and provider/staff levels by 10 and 11 criteria, respectively. The Adoption construct included criteria such as the number and proportion of setting and staff members who agreed to participate in delivering the intervention, description of target locations or providers, how these settings and staff members were recruited, and how representative they were of the intended audience in terms of setting and staff. Implementation was assessed by 11 criteria as our research team added 2 criteria (engagement to inform intervention development and tailoring of intervention). We adapted the tool by including two additional components from the template for intervention description and replication (TIDieR) checklist and guide (15), as these are not covered by RE-AIM: details about tailoring the intervention for participants and the engagement of practitioners, participants, and/or caregivers in the development of the intervention. These components were added to investigate the personalized and tailored nature of health coaching interventions and to reflect our previous systematic review (12) which looked at quadruple aim outcomes beyond the patient level. Other existing criteria included whether interventions were theory-based, detailed descriptions of intervention protocols and how well these protocols were adhered to (fidelity), costs, and the completion rates of intervention participants. Maintenance was evaluated by 8 RE-AIM criteria including sustained impact of the intervention after termination for the participants and at the setting/staff level. See **Supplemental Material 2** for RE-AIM components and definitions of each criteria.

## 3 Results

**Figure 1:**
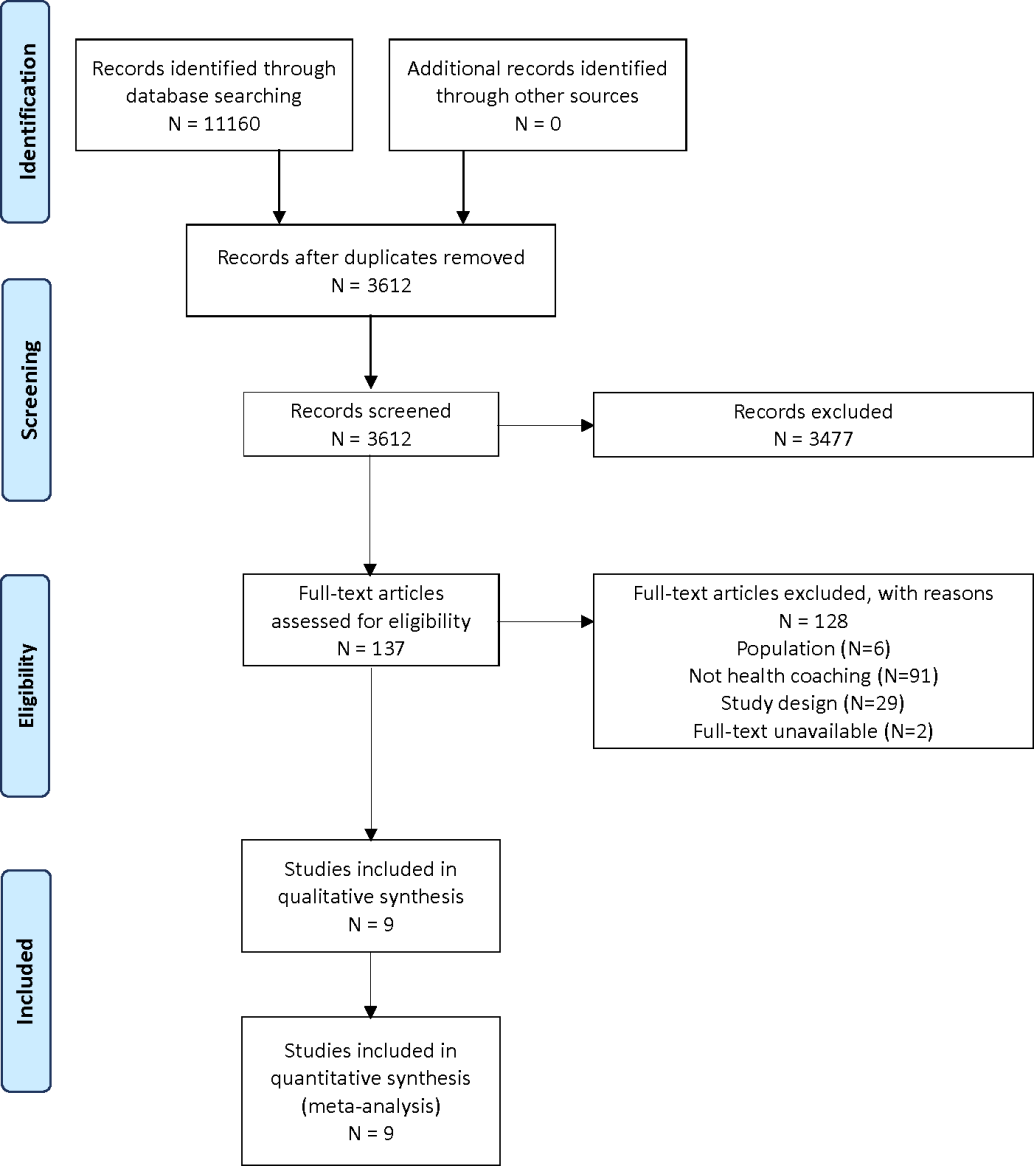
PRISMA flowchart.

From 3,612 citations, we assessed 137 full-text articles for eligibility and included 9 randomized controlled trials (RCTs) (**Figure 1**) (16–24). The studies were published from 2014 to 2021. Studies were conducted across the globe in North America, Europe, and Australia, and intervention duration was between 3 months to 3 years, with most being 6 or 12 months in duration (n=7). Characteristics of the included studies can be found in **Table 1** and further details from studies can be found in **Supplemental Material 3**. A total sample of 2,498 adults with T2D were included in this review with a mean age ranging from 51.0 to 66.6 years and percentage of women in the studies ranging from 10% to 78%. The mean A1C at baseline ranged from 5.5% to 9.9%.

**Table 1:** Characteristics of included studies.

Author, Year	N^1^	Age, mean y (SD)	Gender^2^(% F/M)	Intervention Description	Delivery person(s)	Location/Site of Delivery	Control Description	Study Duration^3^
Balducci, 2019 (16)	300	I: 61.0 (9.7) C: 62.3 (10.1)	39/61	Behavioural intervention through counselling sessions	Certified exercise specialist/diabetologist	Outpatient diabetes clinics	General recommendations for increasing daily physical and decreasing sedentary time	3 years
Cummings, 2019 (22)	139	O: 52.6 (9.6) I: 51.0 (9.0) C: 53.0 (9.0)	78/22	Cognitive behavioral therapy (CBT) plus lifestyle counseling	Team: behavioural providers consisting of a nurse care manager, psychologist, clinical health psychology doctoral student and a community health worker	A large academic family medicine practice	Standard care	12 months
Jutterström, 2016 (20)	327	O: 64.5 (9.58) I: 64.9 (11.10) Internal C: 62.6 (10.61) External C: 66.2 (8.75)	37/63	Group and/or individual sessions to discuss self-management of disease	Diabetes specialist nurses	9 health care centers	Standard care	I: 2-6 months C: 6 months
Karhula, 2015 (24)	287	I: 66.6 (8.2) C: 65.5 (9.6)	44/56	Health coaching over mobile phones and self-monitoring of health parameters with a remote patient monitoring system	Trained personal health coaches	Virtual/phone	Standard care	12 months
Naik, 2019 (19)	225	O: 61.9 (8.3)	10/90	Goal setting for diabetes and depression	Trained health professionals or coaches including psychologists, nurses, pharmacists and social workers	Virtual/phone	Usual care	6 months
Odnoletkova, 2016 (17)	3115	I: 63.8 (8.7) C: 62.4 (8.9)	39/61	COACH model (a continuous quality improvement cycle)	Diabetes nurse educators	Virtual/phone	Usual care	6 months
Sherfali, 2021 (23)	365	I: 56.82 (11.69) C: 59.05 (11.79)	50/50	Diabetes health coaching using case management and monitoring, diabetes self-management education and support with behaviour modification, goal setting and reinforcement in addition to general psychosocial support	Trained registered nurse/certified diabetes educator	Virtual/phone	Usual diabetes education	12 months
Varney, 2014 (21)	94	I: 59 (56–62)^4^ C: 64 (61-66)^4^	32/68	Telephone coaching with encouragement to follow a specified diet and exercise 150 min per week	Dietitian	Virtual/phone	Usual care	6 months
Young, 2020 (18)	319	O: 59.07 (11.4) I: 58.96 (11.3) C: 59.18 (11.5)	47/53	Individual coaching sessions using motivational interviewing to promote mutual goal setting, enhance self-efficacy in health behaviour change, and assist individuals to derive meaning from the data to reinforce choices and behaviours	Registered nurses	Primary care clinics and virtual/phone	Usual care	3 months

O = overall population; I = intervention; C = control.

1. Number of participants randomized at start of study; 2. Values for gender are based on reported baseline which may not equal N randomized but rather the number of participants who completed the intervention; 3. Not including follow-up, if applicable; 4. Reported by the study as mean (95% CI).

There was diversity in how our included studies aligned with definitions and models of diabetes health coaching (6, 9). While all the studies included intervention components related to self-management and education and eight studies also addressed behaviour modification, psychosocial support and case management and monitoring were less common health coaching components. Within these components, studies used a variety of techniques and approaches from general counselling to specialized cognitive behaviour therapy or motivational interviewing. As per our inclusion criteria, all studies used healthcare professionals to deliver the health coaching intervention. For most studies (n=7), just one type of coach was used but in 2 studies (19, 22), a team of health professionals worked together for the delivery of different components of the intervention. Coaches included a certified diabetologist, nurses, psychologists, doctoral students, community health workers, pharmacists, social workers, certified diabetes nurse educators, and a dietitian (**Supplemental Material 3**). Telephone-only strategies were used by 6 studies, while telephone and face-to-face was used in one study, and two studies used in-person or face-to-face strategies only. All the studies were focused on individual or one-on-one interactions and only one study also included group components. Sessions and interactions with the coaches ranged from weekly, to bi-weekly, to as infrequent as one session every 4 to 6 weeks. The duration of these sessions also varied from as short as 15 minutes to as long as 90 minutes; however, most seemed to average around 30 minutes. Any in-person components of the health coaching interventions took place in outpatient healthcare settings such as clinics, healthcare centres, primary care offices, and doctors offices (**Supplemental File 3**).

### 3.1 Overall RE-AIM summary

A summary of the RE-AIM results by each element can be found in **Table 2** (detailed extraction results are available in **Supplemental File 4**). Every study reported on at least one of the 61 RE-AIM criteria; 12 criteria were reported by all 9 included studies and 21 criteria were not reported by any of the studies. Of the 12 criteria reported by all studies, 5 of these were in the Reach element and many are consistent with CONSORT guidelines (25). These criteria include target population, population demographics, inclusion/exclusion criteria for participants, invited participants and sample size numbers, attrition rates, level of expertise of providers, the number, timing, and duration of intervention contacts, and the tailoring of the coaching interventions to individual participant needs. The included studies all reported on more than 20 RE-AIM criteria, ranging from 21 to 27 (**Table 2**). The study that reported the most criteria (27 out of 61) was a one-year RCT which assessed the effectiveness of health coaching over mobile phones and self-monitoring of health parameters with a remote patient monitoring system using trained health coaches (24).

**Table 2:** RE-AIM criteria included in each study.

		Study									
RE-AIM Element	Criteria	Balducci 2019	Cummings 2019	Jutterström 2016	Karhula 2015	Naik 2010	Odnoletkova 2016	Sherifali 2021	Varney 2014	Young 2020	Total
Reach	Described target population	x	x	x	x	x	x	x	x	x	**9**
	Demographic, behavioral information about target population	x	x	x	x	x	x	x	x	x	**9**
	Method to identify the target population	x	x	x	x	x	x		x	x	**8**
	Recruitment strategies	x		x	x	x	x	x	x	x	**8**
	Inclusion/exclusion criteria for individuals	x	x	x	x	x	x	x	x	x	**9**
	Eligible, invited (exposed to recruitment) potential participants	x	x	x	x	x	x	x	x	x	**9**
	Sample size	x	x	x	x	x	x	x	x	x	**9**
	Individual participation rate (sample size/eligible invited potential participants)	x	x	x	x	x	x	x	x	x	**9**
	Comparisons between the target population and the study sample			x	x				x		**3**
	Statistical comparisons between the target population and the study sample			x	x				x		**3**
	Cost of recruitment										**0**
	Qualitative methods to measure reach						x				**1**
Effectiveness	Report of mediators	x	x							x	**3**
	Report of moderators	x	x	x	x			x	x	x	**7**
	Intent-to-treat	x	x		x	x	x	x		x	**7**
	Imputation procedures	x	x		x	x		x	x	x	**7**
	Quality-of-life measures				x		x	x		x	**4**
	Unintended consequences measures/results	x						x			**2**
	Percent attrition (at program completion)	x	x	x	x	x	x	x	x	x	**9**
	Cost-effectiveness										**0**
	Qualitative methods to measure efficacy/effectiveness						x			x	**2**
Adoption, setting	Eligible, invited potential settings			x							**1**
	Number of participating settings	x		x		x					**3**
	Setting participation rate			x							**1**
	Description of the targeted location										**0**
	Inclusion/exclusion criteria of the setting										**0**
	Description of intervention location	x	x	x		x				x	**5**
	Method to identify the setting										**0**
	Comparisons between the targeted and participating settings										**0**
	Statistical comparisons between the targeted and participating settings										**0**
	Average number of persons served per setting										**0**
Adoption, provider/staff	Eligible, invited potential providers (staff)				x						**1**
	Number of participating providers (staff)			x	x	x				x	**4**
	Provider (staff) participation rate				x						**1**
	Method to identify target providers										**0**
	Level of expertise of providers	x	x	x	x	x	x	x	x	x	**9**
	Inclusion/exclusion criteria for providers										**0**
	Comparisons between targeted and participating providers (staff)										**0**
	Statistical comparisons between targeted and participating providers (staff)										**0**
	Measures of cost adoption										**0**
	Dissemination beyond originally planned										**0**
	Qualitative methods to measure adoption						x				**1**
Implementation	Theory-based	x	x	x	x			x		x	**6**
	Engagement to inform intervention										**0**
	Number of intervention contacts	x	x	x	x	x	x	x	x	x	**9**
	Timing of intervention contacts	x	x	x	x	x	x	x	x	x	**9**
	Duration of intervention contacts	x	x	x	x	x	x	x	x	x	**9**
	Extent protocol delivered as intended (fidelity)	x	x		x		x	x			**5**
	Consistency of implementation across settings or providers				x		x			x	**3**
	Tailoring of intervention	x	x	x	x	x	x	x	x	x	**9**
	Participant attendance/completion rates	x	x	x	x	x	x	x	x		**8**
	Measure of intervention cost										**0**
	Qualitative methods to measure implementation										**0**
Maintenance	Follow-up outcome measures at some duration after intervention termination			x		x	x		x	x	**5**
	Attrition/loss to follow-up of individuals			x		x	x		x	x	**5**
	Qualitative methods to measure individual maintenance of the intervention										**0**
	Intervention alignment with the organization’s mission										**0**
	Institutionalization of program after completion of study										
	Maintenance of the program after completion of the study										**0**
	Attrition/loss to follow-up of settings										**0**
	Qualitative methods to measure organizational maintenance/sustainability						x				**1**
**TOTAL FOR STUDY**		**24**	**21**	**26**	**27**	**22**	**25**	**21**	**21**	**26**	

The 'x' means that the variable (row) is present in the Citation (Column). The bold values are to contrast the headings and total counts at the bottom of the table.

### 3.2 RE-AIM criteria

#### 3.2.1 Reach

Reach was the most thoroughly reported RE-AIM construct by the included studies. Eight of the 12 criteria were described by almost all the studies in our review (n=8 or 9). All studies described the target population, provided demographic information about the target population, outlined inclusion/exclusion criteria for screening participants, and provided the number of invited participants, participant rate, and overall sample size of the study. Eight of the 9 included studies also described their methods to identify the target population and their recruitment strategies. Demographic information was not reported consistently across studies, with studies reporting different sample characteristics. For example, while all studies reported on the gender and age of their participants, there was variation in reporting of ethnicity/race (n=5), socioeconomic status (n=5), and chronic diseases/comorbidities (n=4). Only 3 studies compared the target population to their study sample and made statistical comparisons (20, 21, 24). While Jutterstrom et al., and Karhula et al., found no differences in their populations, Karhula et al., did note that those who did not complete the intervention had unfamiliarity with mobile phones. Varney et al., found that their study population was younger and less likely to require an interpreter than the population attending the diabetes clinic from which they recruited. No studies measured the cost of their recruitment and only one study qualitatively measured reach, which was reported in a secondary publication that conducted focus groups and interviews with participants, nurses, and general practitioners (GPs) (17, 26).

#### 3.2.2 Effectiveness

Effectiveness was also well reported by the studies included in our review. All the studies reported on attrition at program completion and most (n=7) reported on moderators, outlined their imputation methods for missing data, and conducted intention-to-treat analysis. Four studies reported on quality of life outcomes and only 2 studies reported adverse events (16, 23). Balducci et al., reported any elective surgeries and medical conditions that occurred outside of the intervention and hypoglycemic episodes, arrythmias, and musculoskeletal injuries or discomfort that occurred during intervention visits or sessions. Sherifali et al., reported on hospitalizations (for any reason), emergency department visits, and hypo- and hyper-glycemic episodes requiring hospitalizations. There were no statistically significant differences in proportion of participants with adverse events between the 2 groups. Two studies used qualitative methods to measure intervention efficacy (17, 18). Both had high rates of participant satisfaction and acceptance with their coaching interventions.

#### 3.2.3 Adoption

Overall, adoption was poorly reported by all studies in our review. While all 9 studies did report the level of expertise of intervention providers, this is likely reflective of our inclusion criteria and selection of studies that used healthcare professionals to deliver the intervention. Five studies did describe the location of the intervention; however, many of our included studies were conducted virtually, *via* the telephone, and therefore did not have a physical intervention location to engage with participants. The rest of the adoption criteria at both the setting and provider/staff level were poorly described as our included studies lacked details about how they selected study locations/settings (eligibility, participation rates, comparisons between settings) and how they selected providers to be involved in intervention delivery (eligibility, participant rates, comparisons between participating and non-participating staff). No studies measured the cost of adoption or if there was dissemination beyond what was originally planned. Only Odnoletkova reported on qualitative methods to measure adoption and found nurses and GPs to be generally accepting and supportive of the intervention (17, 26).

#### 3.2.4 Implementation

Implementation was another well reported RE-AIM construct. All of our included studies reported the number, timing, and duration of intervention contacts (visits or telephone calls) and the tailoring of intervention components to the needs of the participant. This personalization of the intervention is likely reflective of the individual nature of coaching interventions and the fact that our included studies involved mostly one-on-one coaching interactions, rather than group based sessions. Eight of the 9 included studies provided details about participant attendance and completion of the intervention by measuring sessions attended, calls received, and duration of these interactions. Six studies mentioned basing their intervention on a theory or model such as social cognitive theory (16), health belief model (16), cognitive behaviour theory (22), motivational interviewing (18, 23), and others. Fidelity, or the extent the intervention protocol was delivered as intended, was reported by 5 studies using checklists, protocols and manuals, and quality control measures such as supervision or observations by study authors and listening to recordings of interactions between coaches and participants. Three studies also reported on the consistency of implementation across settings and/or providers (different coaches). No studies used any engagement with providers or participants to inform their intervention, and no studies reported on the cost of implementing the intervention or used qualitative methods to measure their implementation.

#### 3.2.5 Maintenance

This construct was poorly reported by all studies in this review. Beyond immediate post-intervention measurements, 5 studies assessed outcomes at a follow-up timepoint and all these studies also reported on the loss of participants during this follow-up period (17–21). Both Jutterstrom et al., and Young et al., did not provide reasons for the loss of participants and the detail provided by Varney et al., for dropouts was vague. Only 1 study used qualitative measures to investigate maintenance and sustainability of such a program (17). No studies assessed or reported on any of the other criteria such as maintenance of the program, modifications made to maintain the program, or alignment of the intervention with the organization’s mission.

## 4 Discussion

This review leverages a recently completed systematic review and meta-analysis examining the effectiveness of diabetes health coaching interventions (12) and examines the application and reporting of the RE-AIM framework to inform future research. Generally, we found good reporting on the reach, effectiveness, and implementation components of the RE-AIM framework, with limited reporting on adoption, and a dearth of reporting on maintenance constructs. The RE-AIM framework was developed to bolster the transparency of reporting of complex interventions, specifically behavioural interventions (13). Ensuring consistent reporting across interventions would lead to an improved understanding of the exact components and implementation of interventions such as diabetes health coaching. However, to date, the application of RE-AIM framework to the diabetes health coaching literature only highlights the gaps in reporting, diversity how these interventions align with health coaching definitions, and exposes limitations in its practical implementation.

From our review of the 9 trials that examined the effectiveness of diabetes health coaching, we found components that addressed the adoption and maintenance criteria were poorly reported. Adoption (e.g. diffusion) relates to the setting and staffing required for the intervention to be deployed. As most studies offered diabetes health coaching virtually (i.e., telephone or technology), it is difficult to ascertain the specific setting-related and staffing requirements that supported the adoption of the intervention. Moreover, the studies were heterogeneous in the descriptions of who could be a health coach (e.g. nurse, physician, exercise physiologist, etc.) and the required training to deliver the coaching intervention (e.g. 120 minutes compared to 8 days of training with credentialed courses). The variability of maintenance, related to the individual or organization implementing diabetes health coaching, was extremely limited in the literature, suggesting that the longer-term impact of diabetes health coaching is not described and has not been evaluated. This corresponds to the limited data on longer term effectiveness of diabetes health coaching beyond 6 months (12), thus making it difficult to understand the impact of diabetes health coaching and the sustained impact of such interventions.

The findings of this review lead to a greater understanding of the evidence and the true impact of interventions, which are behavioural and contextualized to persons and settings. Unfortunately, the evidence related to the implementation of diabetes health coaching and the nature of translating interventions provides gaps in our understanding of and ability translate findings and scale diabetes health coaching interventions to larger populations (27–29). Historically, effectiveness studies and implementation studies have been considered separate entities. Preferably, studies and systematic reviews would be able to report on the effectiveness of the diabetes health coaching intervention and situate the findings within an implementation framework (e.g. RE-AIM), which would better inform stakeholders about practice changes and policies (30).

These latest considerations for merging effectiveness and implementation studies has advanced since the early 2000s, in response to minimizing research waste and the need for bridging the gap from efficacy to effectiveness to implementation into clinical practice (29, 31). With a greater emphasis on effectiveness and implementation focused trials, we will further understand the impact of diabetes health coaching on a variety of health outcomes under ‘usual care’ settings (31). A lack of the studies in this review fulfilling the RE-AIM framework related to diabetes health coaching may suggest that researchers have limited consideration or knowledge of implementation issues when assessing effectiveness of interventions (30). This conceptual incongruency of thinking about “beginning with the end in mind” further perpetuates a delay in uptake and implementation of effective interventions such as diabetes health coaching. Thus, a hybrid approach of effectiveness and implementation designs are only increasing, with the hope that greater transparency and concise reporting with such frameworks as RE-AIM, will evolve the scientific thinking and form a greater appreciation of implementing behavioural interventions like diabetes health coaching in real-world settings.

While our review comprised a comprehensive literature, we did not search grey literature or unpublished industry reports about diabetes health coaching. The exclusion of studies with non-traditional RCT randomization methods may have led to missing implementation trials and thus an under-reporting of studies meeting the Adoption and Maintenance criteria. However, our review leveraged a previous high quality systematic review (11) and we followed rigorous systematic review processes for this update. To this end, this review is a secondary analysis to a recently conducted systematic review and meta-analysis, which explored the effectiveness of diabetes health coaching (12).

## 5 Conclusions

The findings of our review confirm that need for more detailed and transparent reporting related to the implementation of diabetes health coaching. Because of the highly contextualized factors related to behavioural interventions such as diabetes health coaching, it is crucial that research focuses not only on the effectiveness of such interventions but also the implementation. Our review highlights major gaps and a paucity of high-quality evidence related to crucial components of adoption and maintenance of diabetes health coaching. More standardized reporting on external validity is needed to determine whether diabetes health coaching interventions can be effectively delivered, in what setting, by whom it can be delivered, and whether it is sustainable long-term in clinical practice.

## Data availability statement

The original contributions presented in the study are included in the article/**Supplementary Material**. Further inquiries can be directed to the corresponding author.

## Author contributions

All authors were involved in conception and design of the study and approved the protocol; MR, DS were responsible for overseeing the search of databases and literature. MR handled management of database and deduplication or records. MR, MJ, PA, DS were involved in the screening of citations; MR, MJ, PA were responsible for data extraction; MR, DS were responsible for data verification, analysis of data and interpretation of data. All authors supported in the drafting of the manuscript which was led by MR and all authors supported in revising and formatting of the manuscript. All authors have provided final approval of the version of the manuscript submitted for publication, and all authors agree to be accountable for all aspects of the work.

## References

[1] Kimenai DM Pirondini L Gregson J Prieto D Pocock SJ Perel P . Socioeconomic deprivation: An important, largely unrecognized risk factor in primary prevention of cardiovascular disease. Circulation (2022) 146(3):240–8. doi: 10.1161/CIRCULATIONAHA.122.060042 PMC9287096 35748241

[2] Holland SK Greenberg J Tidwell L Malone J Mullan J Newcomer R . Community-based health coaching, exercise, and health service utilization. J Aging Health (2005) 17(6):697–716. doi: 10.1177/0898264305277959 16377768

[3] Kogan AJ . Overcoming obstacles to effective care of type 2 diabetes. Am J Managed Care (2009) 15(9 Suppl):S255–62. 19817514

[4] Hiss RG . The concept of diabetes translation: Addressing barriers to widespread adoption of new science into clinical care. Diabetes Care (2001) 24(7):1293–6. doi: 10.2337/diacare.24.7.1293 11423518

[5] Shah BR Booth GL . Predictors and effectiveness of diabetes self-management education in clinical practice. Patient Educ Counseling (2009) 74(1):19–22. doi: 10.1016/j.pec.2008.08.005 18805668

[6] Wolever RQ Dreusicke M Fikkan J Hawkins TV Yeung S Wakefield J . Integrative health coaching for patients with type 2 diabetes: a randomized clinical trial. Diabetes Educator (2010) 1(4):629–39. doi: 10.1177/0145721710371523 20534872

[7] Huffman M . Health coaching: A new and exciting technique to enhance patient self-management and improve outcomes. Home Healthcare Now (2007) 25(4):271–4. doi: 10.1097/01.NHH.0000267287.84952.8f 17426499

[8] Wong-Rieger D . Health coaching for chronic conditions engaging and supporting patients to self-manage. Toronto, ON: Institute for Optimizing Health Outcomes (2011).

[9] Sherifali D . Diabetes coaching for individuals with type 2 diabetes: A state-of-the-science review and rationale for a coaching model. [Review] (2017) 1(6):547–54. doi: 10.1111/1753-0407.12528 28084681

[10] Pirbaglou M Katz J Motamed M Pludwinski S Walker K Ritvo P . Personal health coaching as a type 2 diabetes mellitus self-management strategy: A systematic review and meta-analysis of randomized controlled trials. Diabetes Educator (2018) 1(7):1613–26. doi: 10.1177/0890117118758234 29658286

[11] Sherifali D Bai JW Kenny M Warren R Ali MU . Diabetes self-management programmes in older adults: a systematic review and meta-analysis. Diabetic Med (2015) 32(11):1404–14. doi: 10.1111/dme.12780 25865179

[12] Racey M Jovkovic M Alliston P Ali MU Sherifali D . Diabetes health coach in individuals with type 2 diabetes: A systematic review and meta analysis of quadruple aim outcomes. Front Endocrinol (2022). 10.3389/fendo.2022.1069401 PMC9800616 36589795

[13] Glasgow RE Vogt TM Boles SM . Evaluating the public health impact of health promotion interventions: the RE-AIM framework. Am J Public Health (1999) 89(9):1322–7. doi: 10.2105/ajph.89.9.1322 PMC1508772 10474547

[14] Harden SM Gaglio B Shoup JA Kinney KA Johnson SB Brito F . Fidelity to and comparative results across behavioral interventions evaluated through the RE-AIM framework: a systematic review. Systematic Rev (2015) 4(1):155. doi: 10.1186/s13643-015-0141-0 PMC4637141 26547687

[15] Hoffmann TC Glasziou PP Boutron I Milne R Perera R Moher D . Better reporting of interventions: template for intervention description and replication (TIDieR) checklist and guide. BMJ Br Med J (2014) 348:g1687. doi: 10.1136/bmj.g1687 24609605

[16] Balducci S D'Errico V Haxhi J Sacchetti M Orlando G Cardelli P . Effect of a behavioral intervention strategy on sustained change in physical activity and sedentary behavior in patients with type 2 diabetes: The IDES_2 randomized clinical trial. JAMA: J Am Med Assoc (2019) 321(9):880–90. doi: 10.1001/jama.2019.0922 30835309

[17] Odnoletkova I Goderis G Nobels F Fieuws S Aertgeerts B Annemans L . Optimizing diabetes control in people with type 2 diabetes through nurse-led telecoaching. Diabetic Med (2016) 33(6):777–85. doi: 10.1111/dme.13092 26872105

[18] Young HM Miyamoto S Dharmar M Tang-Feldman Y . Nurse coaching and mobile health compared with usual care to improve diabetes self-efficacy for persons with type 2 diabetes: randomized controlled trial. JMIR mhealth uhealth (2020) 8(3):e16665. doi: 10.2196/16665 32130184 PMC7076411

[19] Naik AD Hundt NE Vaughan EM Petersen NJ Zeno D Kunik ME . Effect of telephone-delivered collaborative goal setting and behavioral activation vs enhanced usual care for depression among adults with uncontrolled diabetes: a randomized clinical trial. JAMA network Open (2019) 2(8):e198634. doi: 10.1001/jamanetworkopen.2019.8634 31390035 PMC6686779

[20] Jutterström L Hörnsten Å Sandström H Stenlund H Isaksson U . Nurse-led patient-centered self-management support improves HbA1c in patients with type 2 diabetes-a randomized study. Patient Educ Counseling (2016) 99(11):1821–9. doi: 10.1016/j.pec.2016.06.016 27372525

[21] Varney JE Weiland TJ Inder WJ Jelinek GA . Effect of hospital-based telephone coaching on glycaemic control and adherence to management guidelines in type 2 diabetes, a randomised controlled trial. Internal Med J (2014) 44(9):890–7. doi: 10.1111/imj.12515 24963611

[22] Cummings DM Lutes LD Littlewood K Solar C Carraway M Kirian K . Randomized trial of a tailored cognitive behavioral intervention in type 2 diabetes with comorbid depressive and/or regimen-related distress symptoms: 12-month outcomes from COMRADE. Diabetes Care (2019) 42(5):841–8. doi: 10.2337/dc18-1841 30833367

[23] Sherifali D Brozic A Agema P Punthakee Z McInnes N O'Reilly D . Effect of diabetes health coaching on glycemic control and quality of life in adults living with type 2 diabetes: a community-based, randomized, controlled trial. Can J Diabetes (2021) 45(7):594–600. doi: 10.1016/j.jcjd.2020.11.012 33582039

[24] Karhula T Vuorinen AL Rääpysjärvi K Pakanen M Itkonen P Tepponen M . Telemonitoring and mobile phone-based health coaching among Finnish diabetic and heart disease patients: randomized controlled trial. J Med Internet Res (2015) 17(6):e153. doi: 10.2196/jmir.4059 26084979 PMC4526947

[25] Schulz KF Altman DG Moher D . CONSORT 2010 statement: updated guidelines for reporting parallel group randomised trials. BMJ (2010) 340:c332. doi: 10.1136/bmj.c332 20332509 PMC2844940

[26] Odnoletkova I Buysse H Nobels F Goderis G Aertgeerts B Annemans L . Patient and provider acceptance of telecoaching in type 2 diabetes: a mixed-method study embedded in a randomised clinical trial. BMC Med Inf Decision Making (2016) 16:142–. doi: 10.1186/s12911-016-0383-3 PMC5101679 27825340

[27] Tomoaia-Cotisel A Scammon DL Waitzman NJ Cronholm PF Halladay JR Driscoll DL . Context matters: The experience of 14 research teams in systematically reporting contextual factors important for practice change. Ann Family Med (2013) 11(Suppl 1):S115. doi: 10.1370/afm.1549 PMC3707255 23690380

[28] Wolfenden L Foy R Presseau J Grimshaw JM Ivers NM Powell BJ . Designing and undertaking randomised implementation trials: guide for researchers. BMJ (2021) 372:m3721. doi: 10.1136/bmj.m3721 33461967 PMC7812444

[29] Curran GM Bauer M Mittman B Pyne JM Stetler C . Effectiveness-implementation hybrid designs: Combining elements of clinical effectiveness and implementation research to enhance public health impact. Med Care (2012) 50(3):217–26. doi: 10.1097/MLR.0b013e3182408812 PMC3731143 22310560

[30] World Health O . ExpandNet. In: Beginning with the end in mind: planning pilot projects and other programmatic research for successful scaling up. Geneva: World Health Organization (2011).

[31] Wells KB . Treatment research at the crossroads: The scientific interface of clinical trials and effectiveness research. Am J Psychiatry (1999) 156(1):5–10. doi: 10.1176/ajp.156.1.5 9892291

