# Supplementary material for: Applying the RE-AIM implementation framework to evaluate diabetes health coaching in individuals with type 2 diabetes: A systematic review and secondary analysis
Source: Front Endocrinol (Lausanne). 2022 Dec 13;13:1069436. doi: 10.3389/fendo.2022.1069436 (PMC9792599; doi:10.3389/fendo.2022.1069436)
Supplement: Supplementary file 1 [file Table_1.docx]

Supplementary Material

# Search Strategy

MEDLINE

Database: OVID Medline Epub Ahead of Print, In-Process & Other Non-Indexed Citations, Ovid MEDLINE(R) Daily and Ovid MEDLINE(R) 1946 to Present

Search Strategy:

--------------------------------------------------------------------------------

1 diabetes mellitus, type 2/ (149514)

2 type 2 diabetes.tw. (142099)

3 *counseling/ or directive counselling/ (17075)

4 coaching.mp. (7871)

5 "one-on-one".tw. (3598)

6 (individual* adj2 counsel*).tw. (2697)

7 ("face to face" not (face-to-face adj2 interview*)).tw. (20705)

8 telemedicine.mp. (39697)

9 *Internet/ (39124)

10 virtual.mp. (75684)

11 exp cell phone/ or telephone?.mp. (86734)

12 (smart phone? or smart phone? or text messag* or app? or computer application? or phone?).tw. (74837)

13 self-management.mp. (23469)

14 1 or 2 (200685)

15 or/3-13 (344444)

16 14 and 15 (5799)

17 limit 16 to english language (5667)

18 ((random* allocat* or randomi#* or clinical trial? or meta-analy* or metaanaly* or (systematic or quantitative or evidence-based)) adj2 (review? or overview?)).tw. (249056)

19 randomized controlled trial.pt. or randomized controlled trial/ or clinical trial/ or clinical trial.pt. or meta-analysis/ or meta-analysis.pt. or review.pt. (3851915)

20 or/18-19 (3933919)

21 17 and 20 (1896)

22 limit 17 to (comment or editorial or letter or news) (98)

23 21 not 22 (1885)

EMBASE/EMCARE

Database: Embase <1974 to 2021 December 03>

Search Strategy:

--------------------------------------------------------------------------------

1 non insulin dependent diabetes mellitus/ (287559)

2 type 2 diabetes.tw. (216122)

3 1 or 2 (327731)

4 *counseling/ or directive counselling/ (17112)

5 coaching.mp. (10818)

6 "one-on-one".tw. (5222)

7 (individual* adj2 counsel*).tw. (3819)

8 ("face to face" not (face-to-face adj2 interview*)).tw. (27965)

9 telemedicine/ or teleconsultation/ or telemonitoring/ (48259)

10 telemedicine.tw. (19728)

11 *Internet/ (36831)

12 virtual.mp. (100998)

13 exp mobile phone/ (36986)

14 (smart phone? or smart phone? or text messag* or app? or computer application? or phone?).tw. (109419)

15 self-management.mp. (32423)

16 or/4-15 (383152)

17 3 and 16 (7493)

18 limit 17 to english language (7325)

19 ((random* allocat* or randomi#* or clinical trial? or meta-analy* or metaanaly* or (systematic or quantitative or evidence-based)) adj2 (review? or overview?)).tw. (305003)

20 exp randomized controlled trial/ or controlled clinical trial/ (874802)

21 exp meta analysis/ (231649)

22 clinical trial/ or clinical study/ or multicenter study/ or phase 3 clinical trial/ (1381066)

23 or/19-22 (2100116)

24 18 and 23 (2022) EMBASE

EMCARE 976

PSYCINFO

Database: APA PsycInfo <1806 to November Week 5 2021>

Search Strategy:

--------------------------------------------------------------------------------

1 exp type 2 diabetes/ (5167)

2 type 2 diabetes.tw. (7884)

3 1 or 2 (9206)

4 *counseling/ (20072)

5 coaching.mp. (13202)

6 "one-on-one".tw. (3875)

7 (individual* adj2 counsel*).tw. (2785)

8 ("face to face" not (face-to-face adj2 interview*)).tw. (16881)

9 telemedicine/ or teleconsultation/ (6313)

10 telemedicine.tw. (2633)

11 *internet/ (23105)

12 electronic communication/ or social media/ or text messaging/ (15697)

13 virtual.mp. (27815)

14 exp mobile phones/ or mobile devices/ or mobile applications/ (9537)

15 (smart phone? or smart phone? or text messag* or app? or computer application? or phone?).tw. (25690)

16 self-management.mp. (12790)

17 or/4-16 (156529)

18 3 and 17 (1489)

19 limit 18 to english language (1439)

20 ((random* allocat* or randomi#* or clinical trial? or meta-analy* or metaanaly* or (systematic or quantitative or evidence-based)) adj2 (review? or overview?)).tw. (46551)

21 exp Randomized Controlled Trials/ or exp Clinical Trials/ (13053)

22 "systematic review"/ or meta analysis/ (5670)

23 or/20-22 (62481)

24 19 and 23 (90)

COCHRANE

Search Name:

Date Run: 05/12/2021 22:41:48

Comment:

ID Search Hits

#1 MeSH descriptor: [Diabetes Mellitus, Type 2] explode all trees 19238

#2 type 2 diabetes 71126

#3 #1 or #2 71127

#4 MeSH descriptor: [Counseling] this term only 4476

#5 MeSH descriptor: [Directive Counseling] this term only 413

#6 coaching 3944

#7 "one-on-one" 1399

#8 (individual* near/2 counsel*) 1609

#9 ("face to face" not ("face-to-face" near/2 interview*)) 7077

#10 MeSH descriptor: [Telemedicine] explode all trees 3080

#11 telemedicine 4926

#12 MeSH descriptor: [Internet] this term only 4057

#13 "virtual care" or virtual consult* or virtual counsel* or virtual monitor* or "virtual medicine" 3089

#14 MeSH descriptor: [Cell Phone] explode all trees 2103

#15 MeSH descriptor: [Telephone] this term only 2280

#16 "smart phone" or "smart phones" or smartphone* or "text messaging" or "text messages" or "text message" or app or apps or "computer application" or "computer applications" or phone or phones 27584

#17 self-management 9081

#18 MeSH descriptor: [Self-Management] explode all trees 612

#19 #4 or #5 or #6 or #7 or #8 or #9 or #10 or #11 or #12 or #13 or #14 or #15 or #16 or #17 or #18 57962

#20 #3 and #19 5321

702 reviews

4458 trials

CINAHL

| Sunday, December 05, 2021 9:09:56 PM | | | |
| --- | --- | --- | --- |
| **#** | **Query** | **Results** |  |
| S23 | S16 AND S21 | 1,027 |  |
| S22 | S16 AND S21 | 1,067 |  |
| S21 | S17 OR S18 OR S19 OR S20 | 550,655 |  |
| S20 | (MH "Meta Analysis") | 58,983 |  |
| S19 | (MH "Systematic Review") | 103,264 |  |
| S18 | ((random* allocat* or randomi* or clinical trial* or meta- analy* or metaanaly* or (systematic or quantitative or evidence-based)) n2 (review* or overview*)) | 251,270 |  |
| S17 | (MH "Randomized Controlled Trials+") OR (MH "Clinical Trials+") | 329,227 |  |
| S16 | S3 AND S15 | 4,391 |  |
| S15 | S4 OR S5 OR S6 OR S7 OR S8 OR S9 OR S10 OR S11 OR S12 OR S13 OR S14 | 180,878 |  |
| S14 | MH self-management OR self-management | 32,736 |  |
| S13 | smart phone* or smart phone* or text messag* or app or apps or computer application* or phone* | 43,629 |  |
| S12 | (MH "Cellular Phone") OR (MH "Text Messaging") OR (MH "Smartphone") OR (MH "Voice Mail") | 8,659 |  |
| S11 | virtual | 32,293 |  |
| S10 | (MM "Internet") | 27,629 |  |
| S9 | telemedicine | 23,271 |  |
| S8 | (MH "Telemedicine") OR (MH "Remote Consultation") OR (MH "Telenursing") | 18,412 |  |
| S7 | ("face to face" not (face-to-face N2 interview*)). | 9,913 |  |
| S6 | (individual* N2 counsel*) | 1,790 |  |
| S5 | "coaching" | 6,532 |  |
| S4 | (MM "Counseling") | 15,113 |  |
| S3 | S1 OR S2 | 83,091 |  |
| S2 | TX type 2 diabetes | 55,545 |  |
| S1 | (MH "Diabetes Mellitus, Type 2") | 66,477 |  |

# RE-AIM Data Extraction Components and Descriptions

*adapted from Harden SM, Gaglio B, Shoup JA, Kinney KA, Johnson SB, Brito F, et al. Fidelity to and comparative results across behavioral interventions evaluated through the RE-AIM framework: a systematic review. Systematic reviews. 2015;4:155

| **Author, Year, Country** | Self explanatory |
| --- | --- |
| **Target Population** | Brief description of the targeted population (older adults, pre-diabetic women) |
| **Study Setting** | The location(s) where the intervention is delivered.  Example: university/clinical/community/faith-based/home/worksite |
| **Topic Area** | List the health behavior or health topic that is covered (such as cancer, policy, diabetes…) |
| **Purpose** | What is the purpose of the manuscript; provide details that will give us highlights of the paper (e.g., the purpose of this paper was to report on the short term effectiveness of a dietary program, with special attention on the feasibility (adoption) of the program in a clinical setting). |
| **Study Design** | Randomized controlled trail (RCT), clinical controlled trial (CCT), Observational, etc. |
| **Methods used:** | Quantitative, qualitative, review article, meta analysis, narrative piece… |
| **Level/Unit of Analysis:** | Individual, setting, community, combination... |
| **Companion Article** | Is there a companion article to this particular intervention? |
| **Citation** | If so, what is the citation we will need to search (later). |
| **Reach** | The proportion & representativeness of individuals willing to participate in a given intervention |
| **Described target population** | A brief description of the broader target population (i.e., not simply of the study sample).  Example: The target population included all women within the community health center who were over the age of 18 and were not meeting the recommended guidelines for physical activity. |
| **Demographic & behavioral information** | Gender, age, educational attainment, occupation, socioeconomic status, behavioral outcomes. |
| **Method to identify target population** | Describe the process by which the target population was identified for participation in the study.  Example: All patients who were part of the target population were identified using the electronic medical record. |
| **Recruitment Strategies** | Describe the methods used to recruit participants into the study. Example: We used a series of flyers; presentations; mass media; and word of mouth strategies to recruit participants. |
| **Inclusion criteria** | Explicit statement of characteristics of the target population that were used to determine if a potential participant is eligible to participate. Example: The inclusion criteria are… |
| **Exclusion criteria** | Explicit statement of characteristics that would prevent a potential participant from being eligible to participate. Also the percent excluded may be reported.  Example: The exclusion criteria are… |
| **Number Eligible and invited (exposed) to recruitment** | The total number of eligible participants contacted for participation.  Example: 300 people were contacted for the study. After a screener was administered, it was found that of those 300 people contacted, 250 people were eligible. Therefore 250 is the denominator. |
| **Sample size** | The number of people who agree to participate (e.g. n = ) |
| **Participation rate** | Sample size divided by the target population denominator.  Example: 200 (number of people agree to participate)/250 (number of eligible participants contacted for participation) = 80% |
| **Number of Characteristics** | Total number of comparisons made between target population and study sample with a description of what those comparisons were  Example: Participants were compared to non-participants for: activity level, gender, age... |
| **Number of statistically significant comparisons** | Total number of statistically significant comparisons made between target population and study sample with a description of what those comparisons were.  Example: When compared to participants, non-participants were more likely to be older physically inactive females. |
| **Cost of recruitment** | The cost of recruitment can reflect monetary and/or time units.  Example: The overall cost of recruitment strategy A (flyers) was $1000 versus the overall cost of recruitment strategy B (newspaper advertisements) was $200. Could also be coded in cost per participant recruited. |
| **Use of qualitative methods to measure reach** | Reporting on non-quantitative aspects of reach. Observations in words, sentences, descriptions or codes. Some common methods include key informant interviews, focus groups, or even field notes that provide information on perceptions, feelings, opinions, experiences, etc. |
| **Efficacy/Effectiveness** | The influence of an intervention on important outcomes, including potential negative effects, quality of life, & economic outcomes |
| **Report of Mediators** | Variables that explain the extent to which the particular variable accounts for the relationship between the predictor and the criterion. |
| **Report of Moderators** | List of variables: qualitative (e.g., sex, race, class) or quantitative (e.g., level of reward) that influence the direction and/or strength of the relationship between the treatment and the outcome. |
| **Intent-to-treat or present at follow up?** | Intent to treat analysis:  when participants in trials are analyzed in the groups to which they were randomized, regardless of whether they received or adhered to the allocated intervention. Example, will typically use the term intent to treat or will describe an imputation that was used to account for missing data in the analysis.  Present at Follow-up analysis: when only participants who completed the follow-up assessment are included in the analysis of efficacy/effectiveness.  Example: Only those participants who completed both the baseline and follow-up measures were included in the analysis. |
| **Imputation procedures**  **(specify)** | Substitution of some value for missing data.  Example: Multiple imputation methods were used to impute missing minutes of physical activity data at 3 months… |
| **Quality of life measure** | Includes a measure of quality of life with some latitude for coding articles that refer to well-being or satisfaction with life. |
| **Measure unintended consequences (negative) and results** | To evaluate unanticipated consequences and results that may be a product of the intervention and may have caused unintended harm.  Example: In a physical activity promotion program, female participants had an increased rate of injury. |
| **Percent attrition (at program completion)** | The proportion that was lost to follow-up or dropped out of the intervention. This is calculated by dividing the number of participants who did not complete the intervention by the number of participants who began the intervention. ***post intervention** (Not at any follow-up time points); reported total, and by treatment group  Example: 100 participants began the intervention and 20 participants did not complete the intervention. So there was 20% attrition. |
| **Cost effectiveness** | Code as reported if specific mention and amounts are provided for the cost of the intervention.  Example: The new strategy would save $1,000 per life per year when compared to the current practice. |
| **Use of qualitative methods to measure efficacy/effectiveness** | Obtaining qualitative feedback from participants on the degree to which they felt the intervention was efficacious/effective. Some common methods include focus groups, interviews, diaries (text/pictures). |
| **Adoption – Diffusion – Setting Level** | The proportion & representativeness of locations willing to initiate & adopt an intervention |
| **Number eligible and invited (exposed)** | Total sites that met eligibility criteria and were approached for intervention delivery. |
| **Number Participating** | The total number of sites that agreed to participate. |
| **Participation rate** | The proportion of sites eligible and contacted that participated. |
| **Description of targeted location** | Characteristics that would be considered an ideal location for the intervention. |
| **Inclusion/exclusion criteria of setting** | The explicit statement of characteristics of the setting that were used to determine if a potential setting is eligible to participate.  Example: The inclusion/exclusion criteria are... |
| **Description of intervention location** | The explicit statement of characteristics of the location of the intervention.  Example: size of location; resources available staff information; number of eligible locations; work environment/climate |
| **Method to identify setting** | Describe the process by which the location was identified for participation in the study. |
| **Number of Comparisons** | Total number and type of comparisons of targeted intervention sites and those that participated, including a list: size, location, etc. |
| **Number of statistically significant comparisons** | Total statistically significant number and type of comparisons of targeted intervention sites and those that participated, including a list: Schools in urban areas were significantly less likely to agree to participate. |
| **Average Number of persons served per setting** | Calculated average number of participants at each site. |
| **Adoption – Diffusion – Staff Level** | The proportion & representativeness intervention staff willing to initiate & adopt an intervention |
| **Number eligible and invited (exposed)** | Total staff that met eligibility criteria and were approached for intervention delivery. |
| **Number participating in delivery** | The total staff members that agreed to participate. |
| **Participation rate** | The proportion of the staff that was eligible and contacted and participated. |
| **Method to identify target delivery agent** | Describe the process by which the target delivery agent was identified for participation in the study.  Example: All staff at the intervention location that had expertise in leading physical activity classes was identified by supervisors at the intervention location. |
| **Level of expertise of delivery agent** | Training or educational background in relevant area; Degrees, certifications of delivery agents (such as PhD, Masters, Registered Dietitian, etc.) |
| **Inclusion/exclusion criteria of delivery agent** | The explicit statement of characteristics of the delivery agents that were used to determine if a potential delivery agent is eligible to participate.  Example: The inclusion/exclusion criteria are... |
| **Number of Comparisons** | Total number and type of comparisons of targeted staff members and those that participated, including a list: age, body mass index (BMI), education |
| **Number of statistically significant comparisons** | Total statistically significant number and type of comparisons of targeted intervention sites and those that participated, including a list: Staff members who were overweight were less likely to agree to deliver the program. |
| **Measures of cost of adoption** | The price of adoption across all levels of the intervention. At least some mention of start-up (i.e., **not ongoing** just **one time** (start-up)) costs. |
| **Dissemination beyond originally planned** | The spread of the intervention beyond what was planned before the start of the intervention |
| **Use of qualitative methods to measure adoption** | Used qualitative methods to understand the process of adoption.  Example: focus groups, interviews of adoption settings or delivery agents |
| **Implementation** | How consistently various elements of an intervention are delivered as intended by intervention staff, & the time & cost of the intervention |
| **Theories** | Explicit statement of theories or principles used to develop the intervention  Example: social cognitive theory, theory of planned behavior, etc. |
| **Engagement** | Engagement of patient, caregiver, or otherwise, to develop or inform the intervention, post-intervention, etc |
| **Intervention number of contacts** | Total number of encounters with participants for INTERVENTION arm. Could include face-to-face meetings, telephone calls, newsletters etc. *only up to post-intervention (no follow-up visits included) |
| **Timing of contacts** | Describe when the intervention contacts occur over the course of the intervention.  Example: For the first month participants received one telephone call per week and in every month thereafter they received a call a month until the end of the 12 month intervention |
| **Duration of contacts** | Length of each intervention contact. *Direct contact with research personnel, instructor, etc (not just intervention component such as self-led physical activity session)  Example: The first 4 calls lasted about 20 minutes each, the other 11 lasted about 10 minutes each. |
| **Extent protocol delivered as intended** | Description of fidelity to the intervention protocol.  Example: a checklist of program components assessed by delivery agent(s) |
| **Consistency of implementation across setting and delivery agents** | Description of the degree of similarities between multiple settings sites & delivery agents |
| **Tailoring** | If the intervention was planned to be personalized, titrated or adapted, then describe what, why, when, and how. |
| **Participant attendance/completion rates** | The proportion of the intervention that the participants received, on average.  Example: Participants attended 4 of the 6 meetings on average. |
| **Measure of cost** | The **ongoing** cost of delivery across all levels of the intervention |
| **Use of qualitative methods to measure implementation** | Used qualitative methods to understand the process of implementation.  Example: focus groups, interviews |
| **Maintenance** | The extent to which participants make & maintain a behavior change & the sustainability of a program or policy in the setting in which it was intervened |
| **Was individual behavior assessed at some duration following the completion of the intervention? (give duration of follow-up)** | Description of follow-up outcome measures of individuals available at some duration after intervention termination  Example: 6 months after the intervention ended participants had returned to baseline levels of physical activity. |
| **Attrition** | Describe the degree to which participants were lost to follow-up (and the reasons) during the period in time from the interventions completion to the follow-up. |
| **Use of qualitative methods to measure individual maintenance** | Used qualitative methods to understand the process of individual level maintenance (during follow-up period) of changes to the primary outcome.  Example: focus groups, interviews |
| **Report alignment to**  **organization mission** | Was the intervention designed to align with the delivery organization’s mission, values (explicit statement). |
| **Is the program still in place?** | Description of program continuation after completion of the research study. |
| **If no: reason for discontinuation** | Description of why the intervention was terminated |
| **If yes: was the program modified? Specify** | Description of any changes that were made to the original program |
| **Was the program institutionalized?** | Description of the how the intervention was integrated into the delivery system through methods such as policy changes, job description changes. |
| **Attrition** | Describe the degree to which sites were lost to follow-up (and the reasons) during the period in time from the interventions completion to the follow-up. |
| **Use of qualitative methods to measure organizational level maintenance** | Used qualitative methods to understand the process of intervention sustainability at the organizational level |

# Characteristics of Included Studies

Balducci

| Country, Year | Italy, 2019 |
| --- | --- |
| Question/Study Objective | To investigate whether a behavioral intervention strategy can produce a sustained increase in physical activity and reduction in sedentary time among individuals with type 2 diabetes |
| Study Design | Open-label, assessor-blinded, randomized clinical superiority trial |
| Inclusion/Exclusion Criteria | Inclusion: type 2 diabetes (defined by the American Diabetes Association criteria) for at least 1 year, age 40 to 80 years, body mass index of 27 to 40, physical inactivity (ie, insufficient amounts of physical activity according to current guidelines), sedentary lifestyle (ie, >8 hours of time awake spent in a sitting or reclining posture) for at least 6 months, ability to walk 1.6 km without assistance, and clearance by a cardiologist.  Exclusion: NR |
| Sample Size | O: 300, I: 150, C: 150 |
| Loss to Follow-Up | n (%) - O: 33 (11), I: 17 (11.3); C: 16 (10.7) |
| Age | Mean (SD) - I: 61.0 (9.7), C: 62.3 (10.1) |
| Gender | n (%) - Female O: 116 (38.7); I: 59 (39.3); C: 57 (38.0); Male O: 184 (61.3); I: 91 (60.7); C: 93 (62.0) |
| Race/Ethnicity | NR |
| BMI | I: 30.0 (4.9); C: 30.1 (5.3) |
| Duration of Diabetes | Median (IQR) I: 8.0 (4.0-16.0); C: 9.0 (4.0-15.0) |
| Baseline A1C% | Mean (SD) I: 7.4 (1.6); C: 7.3 (1.4) |
| Description of Intervention | Participants in the behavioral intervention group participated in 1 individual theoretical counseling session, conducted by a diabetologist, and 8 biweekly individual theoretical and practical counseling sessions, conducted by a certified exercise specialist, per year for 3 years. |
| Who Delivered Intervention | Certified exercise specialist/diabetologist |
| Description of Coaching Interactions | Frequency: 1 theoretical counselling session, 8 biweekly individual theoretical and practical counselling sessions per year  Duration: NR  Mode: Individual face to face sessions |
| Location/Site of Delivery | Outpatient diabetes clinics |
| Description of Control | Participants in the standard care group received only general physician recommendations for increasing daily physical activity and decreasing sedentary time. |
| Duration of Intervention | I: 3 years; C: 3 years |
| Length of Follow-Up Beyond Post-Intervention | NA |
| Serious Adverse Events | I: 41; C: 59 (hypoglycemia, tachycardia/arrhythmia, musculoskeletal injury/discomfort) |
| Funding Source | Metabolic Fitness Association, Monterotondo, Rome, Italy |

Cummings

| Country, Year | U.S.A., 2019 |
| --- | --- |
| Question/Study Objective | To evaluate the effect of cognitive behavioral therapy (CBT) plus lifestyle counseling in primary care on hemoglobin A1c (HbA1c) in rural adult patients with type 2 diabetes (T2D) and comorbid depressive or regimen-related distress (RRD) symptoms |
| Study Design | Randomized controlled trial |
| Inclusion/Exclusion Criteria | Inclusion: adult patients (18–75 years) with a medical record–established history of T2D with an HbA1c at screening >7.0% (53 mmol/mol) and with a positive screen for symptoms of distress using the Diabetes Distress Scale 2 (DDS-2) item screener (positive = mean score > or = 3 on DDS-2) and/or a positive screen for symptoms of depression on the Patient Health Questionnaire 2 (PHQ-2) item screener (positive = total score > or = 3 on PHQ-2).  Exclusion: Exclusion criteria for screening included a medical record–established diagnosis of advanced disease (e.g., end-stage renal disease, advanced heart failure, blindness, or metastatic cancer) or the presence of alcoholism, cognitive impairment, or major psychiatric illness that would preclude active participation.  If the patient signed consent and met all the screening criteria described above, including having an HbA1c value that day that was >7.0%, then he/she was scheduled for an enrollment visit. |
| Sample Size | O: 139; I: 67; C: 72 |
| Loss to Follow-Up | n (%) - 19 (13.7); I: 10 (13.9); C: 9 (13.4) |
| Age | Mean (SD) – O: 52.6 (9.6); I: 51.0 (9.0); C: 53.0 (9.0) |
| Gender | n (%) – Female O: 108 (77.7); I: 53 (79); C: 55 (76); Male O: 31 (22.3); I: 14 (21); C: 17 (24) |
| Race/Ethnicity | % African American - I: 77%; C: 67% |
| BMI | Weight (lb) Mean (SD) - O: 225 (56.9); I: 217 (57); C: 232 (56) |
| Duration of Diabetes | NR |
| Baseline A1C% | Mean - O: 81; I: 84; C: 79 |
| Description of Intervention | The small-changes lifestyle treatment subgroup included intervention arm patients with low levels of diabetes-related distress and/or depressive symptoms, and the nurse care manager delivered a twice-monthly telephonic intervention focused on lifestyle modifications to improve diabetes and mood based on the small-changes health behavior change model that we have previously described in detail.  The CBT subgroup intervention focused on the reduction of depressive and/or RRD symptoms through modification of negative thoughts and problematic behaviors as well as improvement of diabetes self-management strategies. Sessions were delivered by a clinical health psychologist as well as a doctoral student in clinical health psychology. CBT intervention components were guided by two evidence-based treatment manuals for behavioral activation. Session content used cognitive techniques to identify and challenge general and diabetes-specific cognitive distortions that result in maladaptive behavior, in combination with behavioral techniques, including behavioral activation and specific behavior change strategies related to diabetes and/or mood (self-monitoring, sleep hygiene, eating habits, etc.). These sessions occurred face to face (in the primary care clinic) or via telephonic visits with the health psychologist. Patients who met criteria for the CBT subgroup intervention and yet had more intermediate concerns were provided PST. The PST intervention was based on PST for primary care, which is an adapted version of PST specifically for use in primary care clinics. PST, a variant of CBT, focuses on the facilitation of effective coping and adaptive problem-solving skills and has been shown to be an effective intervention strategy for improvement in diabetes-specific outcomes.  Standard medical care was continued for both arms. However, primary care providers for patients in the intervention arm were offered consultation with a diabetologist to optimize medical management. Primary care providers were asked to titrate medications to appropriate therapeutic dosages based on finger-stick blood glucose response and subsequent HbA1c values. Patient response, adherence, and potential for  side effects were monitored approximately quarterly by the nurse care manager during face-to-face and telephone follow-up, with particular attention to the potential for hypoglycemia associated with insulin and sulfonylurea drugs.  All intervention patients had access to a trained community health worker (CHW) who had extensive experience promoting healthy behaviors for chronic disease management in the targeted region. This CHW provided quarterly telephonic peer support and served as a navigator to community resources that helped patients address logistical challenges to implementing healthy behaviors, problem solving, and accessing healthy food/activity in the target community. |
| Who Delivered Intervention | A team of trained behavioral providers working together, including a nurse care manager who provided small changes lifestyle coaching, a psychologist and clinical health psychology doctoral student who provided CBT sessions including elements of problem-solving therapy (PST), where indicated, and a community health worker (CHW) who provided navigation and social support. |
| Description of Coaching Interactions | Frequency: 1 individual orientation session, 12 individually tailed behavioural treatment sessions  Duration: 30 to 60 minutes  Mode: In-person or optional telephone |
| Location/Site of Delivery | A large academic family medicine practice in the southeastern U.S. |
| Description of Control | Standard medical care |
| Duration of Intervention | I: 12 months; C: 12 months |
| Length of Follow-Up Beyond Post-Intervention | NA |
| Serious Adverse Events | NR |
| Funding Source | East Carolina University |

Jutterström

| Country, Year | Sweden, 2016 |
| --- | --- |
| Question/Study Objective | To evaluate the effect of a patient-centered self-management support, in type 2 diabetes (T2D) with regard to metabolic changes |
| Study Design | Randomized controlled trial with three arms and external control group |
| Inclusion/Exclusion Criteria | Inclusion: diagnosed with T2D within three years, aged 40–80 years, Swedish speaking, and no diagnosed cognitive impairment or other severe illnesses. They had not received patient education other than information given to newly diagnosed T2D patients. |
| Sample Size | O: 327; I: 35; IC: 36; EC: 54 |
| Loss to Follow-Up | n (%) O: 23 (8.5); I: 2 (5.7); IC: 4 (11.1); EC: 7 (13.0) |
| Age | Mean (SD) O: 64.5 (9.58); I: 64.9 (11.10); IC: 62.6 (10.61); EC: 66.2 (8.75) |
| Gender | n (%) Female O: 68 (37.4); I: 13 (38.2); IC: 16 (47.1); EC: 19 (37.3); Male: O: 114 (62.6) I: 21 (61.8); IC: 18 (52.9); EC: 32 (62.7) |
| Race/Ethnicity | NR |
| BMI | O: 30.40 (5.45); I: 31.76 (5.73); IC: 30.56 (5.81); EC: 29.62 (5.27) |
| Duration of Diabetes | NR |
| Baseline A1C% | Mean (SD) O: 5.78 (0.88); I: 5.8 (0.87); IC: 5.8 (0.77); EC: 5.5 (0.84) |
| Description of Intervention | The three groups were group intervention (GI), individual intervention (II), or internal control (IC). All three groups of patients were cared for by the same diabetes nurse at each HCC. Patients randomized to any of the three groups were listed by random and got a number 1, 2, 3, 4, 5, etc.; subsequently they were invited by letter and telephone. The procedure started with an invitation to the first 15 patients in each group and from each HCC.  In the patient intervention, participants in the GI and II groups were invited to six sessions of 45–90 min each, over a period of up to six months. In the GI groups, the patients reflected aspects of living with type 2 diabetes together and the DSNs acted as a moderator, while she acted as the reflective part in the II groups. The content of the intervention is described below. Together with the DSNs, the participants decided when and how often the six sessions should take place, and the intervention period thereby varied between 2 and 6 months. The intervention consisted of either discussions in groups or patients or individual conversations with the DSN, depending on the arm of allocation. During the six sessions, the participants were free to discuss issues they considered important in relation to their experiences with the disease. Each session also had a theme; their views of the image of the illness; the meaning of the diagnosis; the illness integration over time; time and the space for management of the illness in daily life; views on the responsibilities of self-management; and lastly, their prospects for living life with an illness such as medical facts were conveyed only when requested by the participants. The II participants met the local diabetes nurse one-on-one, while the GI participants met in groups where the diabetes nurse acted as a moderator who made sure that everyone in the group participated in the discussion. |
| Who Delivered Intervention | Nine of the nurses had a university education in diabetes care while the tenth had completed diabetes courses offered by pharmaceutical companies. All DSNs participated in a preparatory workshop before the intervention. |
| Description of Coaching Interactions | Frequency: 6 sessions for up to 6 months  Duration: 45-90 minutes  Mode: Face to face |
| Location/Site of Delivery | 9 health care centers in country councils in Northern Sweden |
| Description of Control | An external control group (EC) from an HCC in another county council was recruited with the same inclusion criteria and a similar process for inclusion was used where all patients were listed and one-third of the patients were included. The reason for having an external control group was to control for spillover effects, i.e., unintended positive consequences, which in this case meant that the nurses, when learning a new approach, could also adopt and use the new knowledge on other patients outside the intervention group.  Patients randomized to control groups (IC, EC) received standard care, which normally included 1–2 visits per year according to national guidelines. |
| Duration of Intervention | I: 2-6 months; C: 12 months |
| Length of Follow-Up Beyond Post-Intervention | 5-year study period |
| Serious Adverse Events | NR |
| Funding Source | Strategic Research Programme in Care Sciences, Umeå University and Karolinska Institute, and the Swedish Diabetes Association, the County Council of Västerbotten and Umeå University |

Karhula

| Country, Year | Finland, 2015 |
| --- | --- |
| Question/Study Objective | To study whether a structured mobile phone-based health coaching program, which was supported by a remote monitoring system, could be used to improve the health-related quality of life (HRQL) and/or the clinical measures of type 2 diabetes and heart disease patients |
| Study Design | Randomized controlled trial |
| Inclusion/Exclusion Criteria | Inclusion: diagnosis of type 2 diabetes, glycosylated hemoglobin (HbA1c) level, which needed to be above 6.5% within 1 year prior to the screening, diagnosed with diabetes at least 3 months earlier, 18 years of age or older, ability to fill in questionnaires in Finnish, ability to use the RPM system and the devices provided, having adequate cognitive capacities to participate, being able to walk  Exclusion: NR |
| Sample Size | O: 287; I: 208; C: 79 |
| Loss to Follow-Up | n (%) O: 62 (21.6); I: 46 (22.1); C: 16 (20.3) |
| Age | Mean (SD) – I: 66.6 (8.2); C: 65.5 (9.6) |
| Gender | n (%) - Female O: 111 (44.4); I: 81 (45); C: 30 (43), Male O: 139 (55.6); I: 99 (55); C: 40 (57) |
| Race/Ethnicity | NR |
| BMI | Mean (SD) – I: 31.1 (5.4); C: 30.9 (5.7) |
| Duration of Diabetes | NR |
| Baseline A1C% | Median - I: 7.25; C: 7.20 |
| Description of Intervention | The intervention consisted of health coaching over mobile phones and self-monitoring of health parameters with the help of a remote patient monitoring (RPM) system. A comprehensive evaluation of the patient’s clinical, mental, and social condition was made during the first coaching call and small, achievable health behavior changes were agreed upon with the patient. A self-management plan was created based on the targeted changes. During the mobile phone calls, the health coach provided information, assistance, and support to the patients. The health coaching approach was provided by Pfizer Oy. The approach followed Wagner’s Chronic Care Model—one of the key foundational constructs for the approach of chronic care management—and has been developed and tested earlier. |
| Who Delivered Intervention | Personal health coach trained to obtain the needed knowledge about Pfizer’s health coaching model, behavioral management skills, remote monitoring system, and trial procedures |
| Description of Coaching Interactions | Frequency: Every 4-6 weeks  Duration: 30 minutes  Mode: Telephone |
| Location/Site of Delivery | South Karelia Social and Health Care District (Eksote) |
| Description of Control | Patients assigned to the control group received the care they would have received in the absence of the study. As part of standard care, patients suffering from type 2 diabetes receive a disease management information booklet at the time of diagnosis. Standard care includes laboratory tests taken once a year and 1 appointment or phone call by a nurse or doctor. Patients can contact health care services any time they feel they need to. |
| Duration of Intervention | I: 12 months; C: 12 months |
| Length of Follow-Up Beyond Post-Intervention | NA |
| Serious Adverse Events | NR |
| Funding Source | 50% of the funding of this study was received from the European Commission Information and Communication Technologies Policy Support Program (ICT PSP) 2009 of the Competitiveness and Innovation framework Programme (CIP), as part of the Renewing Health Project involving nine European countries. The other 50% of the funding was provided by Eksote. |

Naik

| Country, Year | U.S.A., 2019 |
| --- | --- |
| Question/Study Objective | To evaluate the effectiveness of proactive population screening plus telephone delivery of a collaborative goal-setting intervention among high-risk patients with uncontrolled diabetes and depression |
| Study Design | Randomized clinical trial |
| Inclusion/Exclusion Criteria | Inclusion: Veterans with uncontrolled diabetes (defined by International Classification of Diseases, Ninth Revision diagnosis code 250.XX and HbA1c of > or =7.5% for 1 year before the study) who lived at least 20 miles from the main Veterans Health Administration hospital in Houston, Texas, or who received primary care services within a MEDVAMC satellite community-based clinic across Southeast Texas.  Exclusion: If there was an absence of depression symptoms, a telephone-based coaching intervention would be inappropriate (eg, the patient had severe cognitive impairment or mental health condition, hearing or visual impairment, or active suicidal ideation), or presence of significant hypoglycemic events or substance abuse. |
| Sample Size | O: 225; I: 136; C: 89 |
| Loss to Follow-Up | n (%) – O: 59 (26.2); I: 40 (29.4); C: 19 (21.3) |
| Age | Mean (SD) – O: 61.9 (8.3) |
| Gender | n (%) – Female O:23 (10.2); I: 15 (11); C: 8 (9); Male O: 202 (89.8); I: 121 (89); C: 81 (91) |
| Race/Ethnicity | n (%) - White - I: 73 (53.7); C: 51 (57.3); non-Hispanic black - I: 41 (30.1); C: 16 (18.0); Hispanic - I: 12 (8.8); C: 11 (12.4); Other - I: 10 (7.4); C: 11 (12.4) |
| BMI | NR |
| Duration of Diabetes | NR |
| Baseline A1C% | Mean (SD) – O: 9.3 (1.4); I: 9.2 (1.4); C: 9.3 (1.5) |
| Description of Intervention | During the first 2 patient sessions, HOPE coaches focused on building rapport, introducing and clarifying values, collaboratively setting initial goals, identifying potential skill sets to address goals, and empowering patients to advocate for their health through active communication with their clinicians. For sessions 3 through 6, participants focused on discrete skill modules (increasing pleasant activities, using thoughts to improve wellness, diet, physical activity, medication management, and relaxation) customized to meet their diabetes and depression goals. Sessions 7 through 9 focused on maintenance skills (reviewing action plans and overcoming barriers). Skills emphasized in the modules were designed to improve diabetes- and depression-related outcomes simultaneously. The HOPE modules stressed the importance of the coach-patient relationship as critical to improvement in participant physical and/or emotional self-management. During months 7 to 12, participants received usual primary care without contact from HOPE coaches. |
| Who Delivered Intervention | Twenty-four trained health professionals or coaches (18 female) included psychologists (n = 16), nurses (n = 5), pharmacists (n = 2), and social workers (n = 1). Most (n = 18) were at the MEDVAMC; 6 were at a Veterans Health Administration community-based clinic |
| Description of Coaching Interactions | Frequency: 9 sessions biweekly from months 1 to 3 and monthly from months 4 to 6; no contact months 7 to 12  Duration: 30-40 minutes from months 1 to 3 and 15 minutes from month 4 to 6  Mode: Telephone |
| Location/Site of Delivery | MEDVAMC and 6 affiliated community-based outpatient clinics across Southeast Texas |
| Description of Control | In addition to usual care, EUC participants were informed about their high-risk status (uncontrolled diabetes status and clinically significant depression symptoms) and were given related educational materials. Study assessments were conducted for EUC participants via telephone, and educational materials were mailed. Participants were encouraged to address these results with their primary care clinician. |
| Duration of Intervention | I: 6 months; C: 6 months |
| Length of Follow-Up Beyond Post-Intervention | 12 months |
| Serious Adverse Events | NR |
| Funding Source | Grant 10-135 from the Veterans Health Administration Health Services Research and Development Office (Drs Cully and Naik) and by grant K23DK11034 from the National Institute of Diabetes and Digestive and Kidney Diseases, National Institutes of Health (Dr Vaughan). |

Odnoletkova

| Country, Year | Belgium, 2016 |
| --- | --- |
| Question/Study Objective | To investigate the effect of the COACH programme on HbA1c and other modifiable diabetes risk factors in people with Type 2 diabetes in a primary care setting in Belgium compared with usual care |
| Study Design | Parallel group, randomized controlled trial |
| Inclusion/Exclusion Criteria | Inclusion: people April 2012 and June 2013, 3115 people were invited to participate in the study. Study participants were adults aged 18–75 years with a diagnosis of Type 2 diabetes, who were receiving glycose-lowering oral and/or injectable therapy.  Exclusion: included corticoid therapy and/or a debilitating coexisting medical condition, such as dialysis, mental illness or cancer; residence in long-term care facilities; pregnancy; and insufficient proficiency in Dutch. |
| Sample Size | O: 3115; I: 287; C: 287 |
| Loss to Follow-Up | n (%) - O: 62 (11); I: 35 (12); C: 27 (9) |
| Age | O: Median of 64 years; I: Mean (SD) 63.8 (8.7); C: Mean (SD) 62.4 (8.9) |
| Gender | Female - O: 221 (38.5); I: 114 (40); C: 107 (37); Male – O: 353 (61.5); I: 173 (60); C: 180 (63) |
| Race/Ethnicity | NR |
| BMI | Mean (SD) - O: 30 (5); I: 30.2 (4.9); C: 30.6 (5.2) |
| Duration of Diabetes | Years (SD) - O: 7 years; I: equal or less than 2 years = 46 (16%); equal or greater 10 years = 94 (33%); C: equal or less than 2 years = 41 (14%); equal or greater 10 years = 91 (32%) |
| Baseline A1C% | Mean (SD) - O: 53 (11); I: 53 (12); C: 53 (11) |
| Description of Intervention | The COACH programme is designed to empower patients to take responsibility for the achievement of their risk factor targets. The coach identifies the ‘treatment gaps’ in the management of each diabetes risk factor, i.e., failure to achieve the guideline-recommended goals, and helps the patient to identify strategies to close the treatment gap, including lifestyle adjustments and adherence to recommended medication therapy. The underlying ‘COACH model’ is a continuous quality improvement cycle, which includes bridging the knowledge gap, assertiveness training, setting an action plan and (re)assessment. The programme consisted of five telephone sessions of a mean (range) duration of 30 (10–45) min, delivered at a mean (range) interval of 5 (3–8) weeks by a certified diabetes nurse educator (hereafter referred to as the ‘coach’) after a 5-day training course. It consisted of an update of the best practice guidelines for the management of Type 2 diabetes, motivational interviewing techniques and software program use. All coaches were employed by a Flemish home care organization, ‘Solidariteit voor het Gezin’. The intervention group received a welcome package containing a nutrition guide, waist circumference metre, BMI calculator and a set for self-monitoring of blood glucose. Participants were instructed on how to perform self-monitoring of blood glucose and interpret the results and were advised on the measurement frequency. They were encouraged to perform the necessary check-ups and to discuss with their general practitioner (GP) drug treatment intensification when appropriate. The coaches analysed patient risk profiles based on the baseline assessment data and consulted GPs on the individual therapeutic goals before the start of the programme. After each session, a written coaching report was prepared by the coach and sent to the participant and his/her GP. The reports contained a comparison between the recommended and the actual outcomes for diabetes risk factors and an agreed action plan to bridge any resulting gap.  The intervention quality control measurements included a review of coaching reports by one of the present authors (I.O.) during the first 3 months and selectively thereafter, audio-recording of several sessions, and weekly programme monitoring briefings. |
| Who Delivered Intervention | Certified diabetes nurse educators after additional training |
| Description of Coaching Interactions | Frequency: 5 monthly telephone sessions  Duration: 30 minutes on average  Mode: Telephone |
| Location/Site of Delivery | Primary care settings |
| Description of Control | The control group received usual care. In Belgium, patients on oral glycaemia-lowering drugs are predominantly treated by their GPs. When insulin therapy needs to be initiated, patients are entitled to a ‘diabetes care trajectory’ that includes diabetes education by a certified diabetes educator and an annual consultation with an endocrinologist, in addition to the regular GP visits. People with advanced diabetes, in need of three or more insulin injections per day, are normally treated by an endocrinologist-led hospital-based diabetes team. All study participants received a DVD with educational material on Type 2 diabetes. The laboratory results of the blood analysis were mailed to all study participants and their GPs. |
| Duration of Intervention | I: 6 months; C: 6 months |
| Length of Follow-Up Beyond Post-Intervention | 18 months |
| Serious Adverse Events | NR |
| Funding Source | The European Regional Development Fund and the Flemish Government. Partena, MSD and Abbott provided a scientific grant for the clinical trial. |

Sherifali

| Country, Year | Canada, 2021 |
| --- | --- |
| Question/Study Objective | To evaluate the effect of a 12-month telephone diabetes health coaching (DHC) intervention on glycemic control in persons living with T2DM |
| Study Design | Community-based randomized controlled trial |
| Inclusion/Exclusion Criteria | Inclusion: > or =18 years of age; b) a T2DM diagnosis (any duration); c) an A1C level >7.5% within 6 months before randomization; d) ability to read and write in English; and e) telephone access  Exclusion: a) pregnancy, b) debilitating coexisting conditions (i.e., mental illness, impaired cognition) and c) underlying medical conditions that could provide misleading A1C levels |
| Sample Size | O: 365; I: 188; C: 177 |
| Loss to Follow-Up | n (%) – O: 8 (2.2%); I: 6 (3.4%); C: 2 (1.1%) |
| Age | Mean (SD) – I: 56.82 (11.69); C: 59.05 (11.79) |
| Gender | n (%) – Female O: 183 (50.1); I: 89 (47.34); C: 94 (53.11); Male O: 182 (49.9); I: 99 (52.66); C: 83 (46.89) |
| Race/Ethnicity | Caucasian % - I: 150 (79.79); C: 144 (81.36) |
| BMI | Mean (SD) – I: 34.71 (7.80); C: 35.36 (8.35) |
| Duration of Diabetes | Years (SD) – I: 10.07 (9.08); C: 9.31 (7.99) |
| Baseline A1C% | Mean (SD) – I: 9.10 (1.65); C: 8.86 (1.50) |
| Description of Intervention | The topic or agenda of each telephone call was determined by the participant or as agreed upon from the previous coaching session. The diabetes health coaching intervention comprised an evidence-informed model of care that included: 1) case management and monitoring; 2) diabetes self-management education and support; 3) behaviour modification, goal setting and reinforcement; and 4) general psychosocial support. The diabetes coaching model philosophy was to provide flexibility and personalization, recognizing that all 4 components are necessary for diabetes self-management and that each component may be required in different amounts and at different times, depending on each participant’s circumstances, goals and needs. |
| Who Delivered Intervention | Registered nurse/certified diabetes educator with training in the DHC model, motivational interviewing and behavioural design, specifically a certification in Tiny Habits coaching, which emphasizes small, positive habits customized to one’s environment, ability and motivation |
| Description of Coaching Interactions | Frequency: Weekly for first 6 months, monthly for last 6 months  Duration: 15 minutes  Mode: Telephone |
| Location/Site of Delivery | Waterloo region of Ontario, Canada |
| Description of Control | All participants in the study received access to usual diabetes education (individual or group) provided by nurses and/or dietitians, typically every 3 to 6 months, along with community resources and an accelerometer. |
| Duration of Intervention | I: 12 months; C: 12 months |
| Length of Follow-Up Beyond Post-Intervention | NA |
| Serious Adverse Events | NR |
| Funding Source | Canadian Institutes of Health Research, Canada (Grant No. 311588) |

Varney

| Country, Year | Australia, 2014 |
| --- | --- |
| Question/Study Objective | To measure the effect of a 6-month telephone coaching intervention on glycaemic control, risk factor status and adherence to diabetes management practices at the intervention’s conclusion (6 months) and at 12 months |
| Study Design | Randomized controlled trial |
| Inclusion/Exclusion Criteria | Inclusion: adults with T2DM and HbA1C >7%  Exclusion: unable to provide consent, non-English speaking, cognitively impaired, receiving palliative care, severely hearing impaired or without telephone access |
| Sample Size | O: 94; I: 47; C: 47 |
| Loss to Follow-Up | O: 6 months: 81 (86.2), 12 months: 71 (75.5); I: 6 months: 9 (19.1), 12 months: 12 (25.5); C: 6 months: 4 (8.5), 12 months: 11 (23.4) |
| Age | Mean (95% CI) - I: 59 (56-62); C: 64 (61-66) |
| Gender | n (%) - Female O: 30 (32.0); I: 13 (28); C: 17 (36), Male O: 64 (68.0); I: 34 (72); C: 30 (64) |
| Race/Ethnicity | n (%) - Caucasian I: 46 (98); C: 37 (79); Asian/Indian I: 1 (2); C: 8 (17); Afro-Caribbean I: 0 (0); C: 2 (4) |
| BMI | Mean (95% CI) - I: 32.1 (30.3-33.9); C: 30.9 (29.1-32.6) |
| Duration of Diabetes | Years (SD) – I: 12.6 (10.2-15.0); C: 13.1 (10.7-15.6) |
| Baseline A1C% | I: 8.2 (8.0-9.7); C: 8.5 (8.1-8.9) |
| Description of Intervention | In addition to usual diabetes care, intervention group participants received 6 months of telephone coaching. Advice given in coaching sessions was consistent with Australian guidelines. During initial coaching sessions, a diet history was taken. Participants were encouraged to follow a low saturated fat, high-fibre diet, with 50% of energy from carbohydrates, and were encouraged to exercise for 150 min per week. Risk factor status and adherence to monitoring requirements were based on information collected at baseline. For treatment goals and risk factors not at target levels, the dietary, lifestyle and medication changes required to improve these parameters were discussed. The coach delivering the intervention did not prescribe medication, therefore, participants were advised to discuss medication changes with their general practitioner (GP). Discrepancies between participants’ adherence to self-care activities (diet and physical activity) and monitoring requirements (foot checks, eye checks and vaccinations) were highlighted and the appropriate management schedule was explained. Participant goals were then agreed, be this a change in diet or a podiatry appointment for an overdue foot examination. Following each coaching session, the participant and their GP received a letter summarising the participant’s goals.  During subsequent coaching sessions, progress towards treatment goals, risk factor status, adherence to self-care and monitoring requirements were reassessed. If goals were not achieved, barriers to goal attainment were identified, an action plan addressing these barriers was agreed and new goals were established. This process was repeated throughout the intervention. |
| Who Delivered Intervention | Dietitian with experience in cardiovascular disease and T2D |
| Description of Coaching Interactions | Frequency: Monthly, mean number of sessions 6.0 (range 4-9)  Duration: Flexible, determined by time required to establish participant goals. Typically, initial and follow-up sessions took 45 and 20 minutes respectively.  Mode: Telephone |
| Location/Site of Delivery | Diabetes Clinic of St Vincent’s Hospital Melbourne (STV), an Australian public teaching hospital |
| Description of Control | Controls did not receive the telephone coaching intervention, or any contact from the researchers, with the exception of telephone calls to arrange baseline, 6- and 12-month assessment appointments. Control group participants could access STV usual care services, including a diabetes clinic staffed by endocrinologists, diabetes educators and dietitians. STV patients typically attend the diabetes clinic 3–6 monthly, with GP visits occurring at the patient’s discretion. |
| Duration of Intervention | I: 6 months; C: 6 months |
| Length of Follow-Up Beyond Post-Intervention | 12 months |
| Serious Adverse Events | NR |
| Funding Source | St Vincent’s Hospital, Research Endowment Fund |

Young

| Country, Year | U.S.A., 2020 |
| --- | --- |
| Question/Study Objective | To evaluate the effectiveness of a nurse coaching program using motivational interviewing paired with mobile health (mHealth) technology on diabetes self-efficacy and self-management for persons with type 2 diabetes |
| Study Design | Randomized controlled trial |
| Inclusion/Exclusion Criteria | Inclusion: (1) aged 18 years or above, (2) receiving care at 1 of the 3 clinics, (3) living with type 2 diabetes and having HbA1c of 6.5% (48 mmol/mol) or higher, and (4) able to speak English  Exclusion: no access to a telephone, were not able to consent because of cognitive impairment, or were pregnant |
| Sample Size | O: 319 (392 invited); I: 158; C: 161 |
| Loss to Follow-Up | n (%) – O: 32 (10.0); I: 26 (16.5); C: 6 (3.7) |
| Age | Mean (SD) – O: 59.07 (11.4); I: 58.96 (11.3); C: 59.18 (11.5) |
| Gender | n (%) – Female O: 148 (47.3); I: 73 (47.4); C: 75 (47.2); Male O: 165 (52.7); I: 81 (52.6); C: 84 (52.8) |
| Race/Ethnicity | Race n (%) - Caucasian I: 96 (63.2); C: 100 (62.9), African American I: 21 (13.8); C: 18 (11.3), Asian I: 11 (7.2); C: 16 (10.1), Other I: 16 (10.5); C: 14 (8.8), More than 1 race I: 8 (5.3); C: 11 (6.9)  Ethnicity n (%) - Hispanic or Latino I: 24 (17.5); C: 18 (12.9), Not Hispanic or Latino I: 113 (82.5); C: 122 (87.1) |
| BMI | NR |
| Duration of Diabetes | NR |
| Baseline A1C% | NR |
| Description of Intervention | We paired each participant with a nurse health coach who delivered 6 individual sessions using a counseling style based on the concepts of MI. Sessions were structured to promote mutual goal setting, enhance self-efficacy in health behaviour change, and assist individuals to derive meaning from the data to reinforce choices and behaviours. Two RN researchers with nurse coaching experience in diabetes audited 8 of the 158 (5%) of the participant sessions and scored the coach using the MITI. They provided timely feedback to the coaches during weekly debriefing sessions, reviewed scores, and discussed optimization strategies by reviewing scenarios.  The initial MI session elicited motivations and set goals with tracking metrics to gauge the progress toward goals at subsequent sessions. Throughout the sessions, the coaches encouraged the participants to identify facilitators and barriers to achieving their health goals. |
| Who Delivered Intervention | Nurse health coaches for the intervention were 3 registered nurses (RNs) with experience in both health coaching and management of chronic disease |
| Description of Coaching Interactions | Frequency: Every 2 weeks for 3 months  Duration: NR  Mode: In-person orientation followed by telephone sessions |
| Location/Site of Delivery | Primary care clinics in Northern California |
| Description of Control | Participants in this group received usual care through their primary care clinic. Usual care comprised standard health care visits with providers and access to classes, resources, and services (i.e., diabetes management and weight loss education, electronic learning videos, and care coordination). At the orientation meeting, the study team members provided instruction on how to access these resources and services as well as how to use the health system’s patient portal (MyChart). |
| Duration of Intervention | I: 3 months; C: 3 months |
| Length of Follow-Up Beyond Post-Intervention | 9 months |
| Serious Adverse Events | NR |
| Funding Source | Patient-Centered Outcomes Research Institute: IHS-1310-07894 |
